# Unfractionated heparin and enoxaparin reduce high-stretch ventilation augmented lung injury: a prospective, controlled animal experiment

**DOI:** 10.1186/cc7949

**Published:** 2009-07-06

**Authors:** Li-Fu Li, Chung-Chi Huang, Horng-Chyuan Lin, Ying-Huang Tsai, Deborah A Quinn, Shuen-Kuei Liao

**Affiliations:** 1 Department of Medicine, Division of Pulmonary and Critical Care Medicine, Chang Gung Memorial Hospital, 5 Fu-Hsing Street, Kweishan, Taoyuan 333, Taiwan, Republic of China; 2 Chang Gung University, 259 Wen-Hwa 1st Road, Kweishan, Taoyuan 333, Taiwan, Republic of China; 3 Department of Respiratory Therapy, Chang Gung Memorial Hospital, 5 Fu-Hsing Street, Kweishan, Taoyuan 333, Taiwan, Republic of China; 4 Department of Medicine, Pulmonary and Critical Care Units, Massachusetts General Hospital, 55 Fruit Street, Bulfinch 148, Boston, MA 02114, USA; 5 Harvard Medical School, 25 Shattuck Street, Boston, MA 02115, USA; 6 Novartis Institute of Biomedical Research, 250 Massachusetts Avenue, Cambridge 02140, MA, USA; 7 Graduate Institute of Clinical Medical Sciences, Chang Gung University, 259 Wen-Hwa 1st Road, Kweishan, Taoyuan 333, Taiwan, Republic of China

## Abstract

**Introduction:**

Dysregulation of coagulation and local fibrinolysis found in patients with acute lung injury often results in the need for the support of mechanical ventilation. High-tidal-volume mechanical ventilation can increase lung damage and suppression of fibrinolytic activity, but the mechanisms are unclear. We hypothesized that subcutaneous injections of unfractionated heparin and enoxaparin would decrease neutrophil infiltration, lung edema, and plasminogen-activator inhibitor-1 (PAI-1) production in mice exposed to high-tidal-volume ventilation.

**Methods:**

Male C57BL/6 mice, weighing 20 to 25 g, were exposed to either high-tidal-volume (30 ml/kg) or low-tidal-volume (6 ml/kg) mechanical ventilation with room air for 1 to 5 hours after 200 IU/kg or 400 IU/kg unfractionated heparin and 4 mg/kg or 8 mg/kg enoxaparin administration. Nonventilated mice served as a control group. Evan blue dye, lung wet- to dry-weight ratio, histopathologic grading of epithelium, myeloperoxidase, and gene expression of PAI-1 were measured. The expression of PAI-1 was studied by immunohistochemistry.

**Results:**

High-tidal-volume ventilation induced increased microvascular permeability, neutrophil influx, PAI-1 mRNA expression, production of PAI-1 protein, and positive staining of PAI-1 in epithelium in a dose-dependent manner. Lung injury induced by high-tidal-volume ventilation was attenuated with PAI-1-deficient mice and pharmacologic inhibition of PAI-1 activity by low-dose unfractionated heparin and enoxaparin.

**Conclusions:**

We conclude that high-tidal-volume mechanical ventilation increased microvascular permeability, neutrophil influx, lung PAI-1 mRNA expression, production of active PAI-1. The deleterious effects were attenuated by low-dose unfractionated heparin or enoxaparin treatment. Understanding the protective mechanism of unfractionated heparin and enoxaparin related to the reduction of PAI-1 may afford further knowledge of the effects of mechanical forces in the lung and development of possible therapeutic strategies involved in acute lung injury.

## Introduction

Acute respiratory distress syndrome (ARDS) is an inhomogeneous lung disease characterized by neutrophil influx into the lungs, increased expression of inflammatory cytokines or chemokines, loss of epithelial and endothelial integrity, and the development of alveolar and interstitial pulmonary edema [1]. The use of high tidal volume in normal animals mimics this overdistention of the normal lung. Mechanical ventilation with high tidal volumes (V_T_) causes acute lung injury (VILI, ventilator-induced lung injury) characterized by an inflammatory response that is morphologically and histologically similar to that caused by bleomycin or bacterial lipopolysaccharide [2,3]. High-tidal-volume ventilation can lead to the production of inflammatory cytokines, including plasminogen activator inhibitor-1 (PAI-1), transforming growth factor-β1 (TGF-β1), and murine macrophage inflammatory protein-2 (MIP-2); apoptosis of airway epithelial cells; lung neutrophil influx; and capillary leak [4,5].

Intraalveolar fibrin formation in acute lung injury occurs after capillary alveolar leakage of plasma fibrinogen, activation of coagulation, and suppression of local fibrinolysis, activating endothelial cells to produce proinflammatory mediators, and eliciting recruitment and activation of neutrophils. Local production of PAI-1 has been found to suppress the fibrinolytic activity in bronchoalveolar fluid (BAL) from patients with ARDS supported by high-tidal-volume mechanical ventilation [6]. PAI-1, a member of the serine protease inhibitor (serpin) gene family, rapidly inhibits both urokinase-type plasminogen activator (uPA) and tissue-type plasminogen activator (tPA) [7]. PAI-1 also forms complexes with other serine proteases involved in the coagulation cascade, including factors Xa, XIa, XIIa, kallikrein, and thrombin, especially in the presence of cofactors of heparin or vitronectin [8].

Enoxaparin, one of the low-molecular-weight heparins (LMWHs), is safer and easier to administer than unfractionated heparin, a naturally occurring glycosaminoglycan with both anticoagulant and antiinflammatory activities [9]. LMWH was found in a porcine acute lung injury model to have protective effects because of its effects on neutrophil emigration, edema formation, tumor necrosis factor-α (TNF-α), and thromboxane B_2 _production [10,11]. Previous studies showed the protective effects of heparin in animal models of lung injury [9,10]. In this high-tidal-volume ventilation-induced acute lung injury model in mice, we compare the effects between different doses of low-molecular-weight and unfractionated heparin, and correlation of acute lung injury to production of PAI-1 by using animals deficient in PAI-1. We hypothesized that subcutaneous injections of unfractionated heparin or enoxaparin would decrease neutrophil infiltration, lung edema, and PAI-1 production in mice exposed to high-tidal-volume ventilation.

## Materials and methods

### Experimental animals

Male C57BL/6, either wild-type PAI-1^+/+ ^or PAI-1^-/- ^on a C57BL/6 background, aged between 6 and 8 weeks, weighing between 20 and 25 g, were obtained from Jackson Laboratories (Bar Harbor, ME, USA) and the National Laboratory Animal Center (Taipei, Taiwan). Mice that are homozygous for the targeted mutation are viable and fertile and do not display any gross behavioral abnormalities. The disruption of the *Serpine1 *gene induces a mild hyperfibrinolytic state. Compared with that in wild-type mice, pulmonary clot lysis is increased in the homozygote. Endotoxin-induced venous thrombosis is decreased compared with that in wild-type mice [12]. The construct PAI-1, containing a PGK-neomycin resistance cassette, replaced all of the *serpine1 *coding sequence and part of the promoter region, including the transcription-initiation site and is electroporated into 129S2/SvPas-derived E3 embryonic stem (ES) cells. Injection of the ES cells into C57BL/6 (B6) blastocysts generated chimeras. The resulting chimeric male animals were crossed to C57BL/6 mice, and then backcrossed to the same for eight generations. The lower expressions of the PAI-1 protein in PAI-1^-/- ^mice was confirmed by using Western blot analysis. The study was performed in accordance with the animal experimental guidelines of the National Institutes of Health and with the approval of the local research committee.

### Experimental groups

The experimental group of animals and procedures used in this study is summarized in Table 1.

**Table 1:** Experimental groups and procedure of animals per group

	EBD (5 hours)	Lung water, PAI-1 (5 hours)	Neutrophils, MPO (5 hours)	PAI-1 mRNA (1 hour)	IHC, H&E stain (5 hours)
Control	6	6	6	6	6
V_T_6 ml/kg	6	6	6	6	6
V_T_30 ml/kg	6	6	6	6	6
V_T_30 ml/kg + 200 IU/kg UFH	6	6	6	6	6
V_T_30 ml/kg + 400 IU/kg UFH	6	6	6	6	6
V_T_30 ml/kg + 4 mg/kg enoxaparin	6	6	6	6	6
V_T_30 ml/kg + 8 mg/kg enoxaparin	6	6	6	6	6
V_T_30 ml/kg + PAI-1^-/-^	6	6	6	6	6

Control = spontaneously breathing, nonventilated mice; EBD = Evans blue dye, a measurement of microvascular leak in the lung; H&E = hematoxylin and eosin; IHC = immunohistochemical stain; lung water = measured by lung wet- to dry-weight ratio; MPO = myeloperoxidase, a measurement of total neutrophil infiltration in the lung; PAI-1 = plasminogen-activator inhibitor-1; UFH = unfractionated heparin; V_T _= tidal volume. Time points for measurements were determined according to our previous findings that mediator activation occurs early in ventilator-induced lung injury, and neutrophil infiltration occurs later [14].

### Ventilator protocol

We used our established mouse model of VILI, as previously described [13,14]. A 20-gauge angiocatheter was introduced into the tracheotomy orifice of mice under general anesthesia with intraperitoneal ketamine (90 mg/kg) and xylazine (10 mg/kg). The mice were placed in a supine position on a heating blanket and then attached to a Harvard apparatus ventilator, model 55–7058 (Harvard Apparatus, Holliston, MA, USA), set to deliver either 6 ml/kg at a rate of 135 breaths per minute or 30 ml/kg at a rate of 65 breaths per minute, for 1 and 5 hours while breathing room air with zero end-expiratory pressure. The mice then received 0.9% saline containing maintenance ketamine (0.1 mg/g/h) and xylazine (0.01 mg/g/h) at a rate of 0.09 ml/10 g/h by a continuous intraperitoneal fluid pump. The tidal volume delivered by the ventilator was checked by fluid displacement from an inverted calibration cylinder. Continuous monitoring of end-tidal CO_2 _by a microcapnograph (Columbus Instruments, Columbus, OH, USA) was performed, and respiratory frequencies of 135 breaths per minute for 6 ml/kg and 65 breaths per minute for 30 ml/kg were chosen in the experiment, with end-tidal CO_2 _at 30 to 40 mm Hg. Airway peak inspiratory pressure was measured with a pressure-transducer amplifier (Gould Instrument Systems, Valley View, OH, USA) connected to the tubing at the proximal end of the tracheostomy. Mean arterial pressure was monitored every hour during mechanical ventilation by using the same pressure-transducer amplifier connected to a 0.61-mm outer diameter (0.28-mm inner diameter) polyethylene catheter ending in the common carotid artery. At the end of the study period, heparinized blood was taken from the arterial line for analysis of arterial blood gas, and the mice were killed. Control, nonventilated mice were anesthetized and killed immediately.

### Unfractionated heparin and enoxaparin administration

Unfractionated heparin (Sigma, St. Louis, MO, USA), 200 IU/kg or 400 IU/kg, and enoxaparin (Sigma), 4 mg/kg or 8 mg/kg, were given subcutaneously 30 minutes before ventilation, based on previous *in vivo *studies that showed that 200 IU/kg heparin and 4 mg/kg enoxaparin inhibited blood coagulation and lung injury [9,15].

### Evans Blue dye analysis

Extravasation of Evans blue dye (EBD; Sigma) into the interstitium was used as a quantitative measure of changes of microvascular permeability in acute lung injury [13]. Thirty minutes before the end of mechanical ventilation, 30 mg/kg of Evans blue dye was injected through the internal jugular vein. At the time of death after 5 hours of mechanical ventilation, the lungs were perfused, free of blood, with 1 ml of 0.9% normal saline *via *the right ventricle and removed *en bloc*. Evans blue was extracted from lung tissue after homogenization for 2 minutes in 5 ml of formamide (Sigma) and incubated at 37°C overnight. The supernatant was separated by centrifugation at 5,000 *g *for 30 minutes, and the amount was recorded. Evans blue in the plasma and lung tissue was quantitated by dual-wave-length spectrophotometric analysis at 620 and 740 nm. The method corrects the specimen's absorbance at 620 nm for the absorbance of contaminating heme pigments, by using the following formula:

We calculated the Evans blue dye amount extracted from lung tissue and divided the amount by the weight of the lung tissue.

### Analysis of lung water

Lungs were removed *en bloc*, and large airways were removed. Both lungs were weighed and then dried in an oven at 80°C for 48 hours. If no changes were found in the dry lung weight at 24 and 48 hours, the weight at 48 hours was used. Lung wet- to dry-weight ratio was used as an index of pulmonary edema formation [16].

### Histopathologic grading of VILI

The lung tissues from control, nonventilated mice and mice exposed to high- or low-tidal-volume ventilation for 5 hours while breathing room air were removed *en bloc *and filled with 10% neutral buffered formalin (pH 6.8 to 7.2) at 30-cm H_2_O pressure *via *polyethylene tubing inserted into the trachea. The lungs were paraffin embedded, sliced at 4 μm, stained with hematoxylin and eosin, and reviewed from 10 nonoverlapping fields by a single investigator blinded to the mouse genotype. Lung injury was scored by using the average of the following items: alveolar congestion, hemorrhage, infiltration of neutrophils into airspace or the vessel wall, and thickness of the alveolar wall [17]. A score of 0 represented normal lungs; 1, mild, <25% lung involvement; 2, moderate, 25% to 50% lung involvement; 3, severe, 50% to 75% lung involvement; and 4, very severe, >75% lung involvement.

### Cell counts

Neutrophil counts were used to measure migration of neutrophils into the alveoli, as previously described [13]. Total cell counts in lung-lavage fluid were performed by using a hemocytometer. To perform cell differentials, cells were fixed on glass slides by using cytospin and stained with geimsa.

### Myeloperoxidase assay

The lungs (0.12 to 0.17 g) were homogenized in 5 ml of phosphate buffer (20 mmol/L, pH 7.4). One milliliter of the homogenate was centrifuged at 10,000 *g *for 10 minutes at 4°C. The resulting pellet was resuspended in 1 ml of phosphate buffer (50 mmol/L, pH 6.0) containing 0.5% hexadecyltrimethylammonium bromide. The suspension was then subjected to three cycles of freezing (on dry ice) and thawing (at room temperature), after which it was sonicated for 40 seconds and centrifuged again at 10,000 *g *for 5 minutes at 4°C. The supernatant was assayed for MPO activity by measuring the hydrogen peroxide (H_2_O_2_)-dependent oxidation of 3,3', 5,5'-tetramethylbenzidine (TMB). In its oxidized form, TMB has a blue color, which was measured spectrophotometrically at 650 nm. The reaction mixture for analysis consisted of a 25-μl tissue sample, 25 μl of TMB (final concentration, 0.16 mmol/L) dissolved in dimethylsulfoxide, and 200 μl of H_2_O_2 _(final concentration, 0.30 mmol/L) dissolved in phosphate buffer (0.08 mol/L, pH 5.4). The reaction mixture was incubated for 3 minutes at 37°C, and the reaction was stopped by adding 1 ml of sodium acetate (0.2 mol/L, pH 3.0), after which absorbance at 650 nm was measured. The absorbance (A650) was reported as optical density (OD)/g of wet lung weight [13].

### Measurement of PAI-1

At the end of the study period, the lungs were lavaged *via *tracheostomy with a 20-gauge angiocatheter (sham instillation) 3 times with 0.6 ml of 0.9% normal saline. The effluents were pooled and centrifuged at 2,000 rpm for 10 minutes. Supernatants were frozen at -80°C for further analysis of the cytokine. PAI-1 with a lower detection limit of 0.02 ng/ml was measured in serum and BAL fluid by using a commercially available immunoassay kit containing primary polyclonal anti-mouse antibody that was cross-reactive with rat and mouse PAI-1 (Molecular Innovations, Inc., Southfield, MI, USA). Each sample was run in duplicate according to the manufacturer's instructions.

### Reverse transcription-polymerase chain reaction (RT-PCR)

For isolating total RNA, the lung tissues were homogenized in TRIzol reagents (Invitrogen Corporation, Carlsbad, CA, USA) according to the manufacturer's instructions. Total RNA (1 μg) was reverse transcribed by using a GeneAmp PCR system 9600 (PerkinElmer, Life Sciences, Inc., Boston, MA, USA), as previously described [18]. The following primers were used for PAI-1: forward primer 5'-TCAGAGCAACAAGT TCAACTACACTGAG-3'and reverse primer 5'-CCCACTGTCAAGGCTCCATCA CTTGCCCCA-3', and glyceraldehyde-phosphate dehydrogenase (GAPDH) as internal control by using the following primers: forward primer 5'-AATGCATCCTGCA CCACCAA-3' and reverse primer 5'-gtagccatattcattgtcata-3' (Integrated DNA Technologies, Inc., Coralville, IA, USA) [19].

### Immunohistochemistry

The lung tissues from control, nonventilated mice and mice exposed to high-tidal-volume ventilation for 5 hours while breathing room air were paraffin embedded, sliced at 4 μm, deparaffinized, antigen unmasked in 10 mmol/L sodium citrate (pH 6.0), incubated with rabbit anti-PAI-1 primary antibody (1:100; New England BioLabs, Beverly, MA, USA) and biotinylated goat anti-rabbit secondary antibody (1:100) according to the manufacturer's instruction for an immunohistochemical kit (Santa Cruz Biotechnology). The specimens were further conjugated with horseradish peroxidase/streptavidin complex, detected with a diaminobenzidine (DAB) substrate mixture, and counterstained by hematoxylin. A dark-brown DAB signal, identified by arrows, indicated positive staining of PAI-1 of epithelial cells, whereas shades of light blue signified nonreactive cells.

### Statistical evaluation

The PAI-1 mRNAs were quantitated by using a National Institutes of Health (NIH) image analyzer, ImageJ 1.27z (National Institutes of Health, Bethesda, MD, USA) and presented as arbitrary units. Values were expressed as the mean ± SD for at least six experiments. The data of lung wet- to dry-weight ratio, total cell counts, neutrophils, PAI-1, MPO, and Evans blue dye were conducted by using Statview 5.0 (Abascus Concepts, Inc., Cary, NC, USA; SAS Institute, Inc.). All results of PAI-1 mRNA and MPO were normalized to control, nonventilated mice breathing room air. ANOVA was used to assess the statistical significance of the differences, followed by multiple comparisons with a Scheffé test, and a *P *value < 0.05 was considered statistically significant.

## Results

### Physiological data

No statistical difference was found in pH, PaO_2_, PaCO_2_, mean arterial pressure, and peak inspiratory pressure at the beginning versus the end of 5 hours of mechanical ventilation (Table 2).

**Table 2:** Physiological conditions at the beginning and end of ventilation

	Nonventilated Room air	V_T _6 ml/kg Room air	V_T _30 ml/kg Room air
PH	7.41 ± 0.04	7.36 ± 0.03	7.32 ± 0.05
PaO_2 _(mm Hg)	99.1 ± 0.4	82.6 ± 7.3	86.8 ± 1.2
PaCO_2 _(mm Hg)	40.3 ± 0.4	42.1 ± 1.5	35.1 ± 1.7
MAP (mm Hg)			
Start	87 ± 1.6	85.7 ± 2.6	84.2 ± 2.1
End	85 ± 0.7	81.2 ± 1.9	73.6 ± 6.3
PIP, mm Hg			
Start		9.7 ± 1.3	23.8 ± 2.5
End		11.8 ± 1.6	28.1 ± 3.4

Arterial blood gases and mean arterial pressure of normal nonventilated mice and mice ventilated at a tidal volume of 6 ml/kg or 30 ml/kg for 5 hours (n = 10 per group). V_T _= tidal volume; MAP = mean arterial pressure; PIP = peak inspiratory pressure.

### Inhibition of high-tidal-volume–induced microvascular leak, lung edema, and total lung injury with unfractionated heparin and enoxaparin

**Figure 1:**
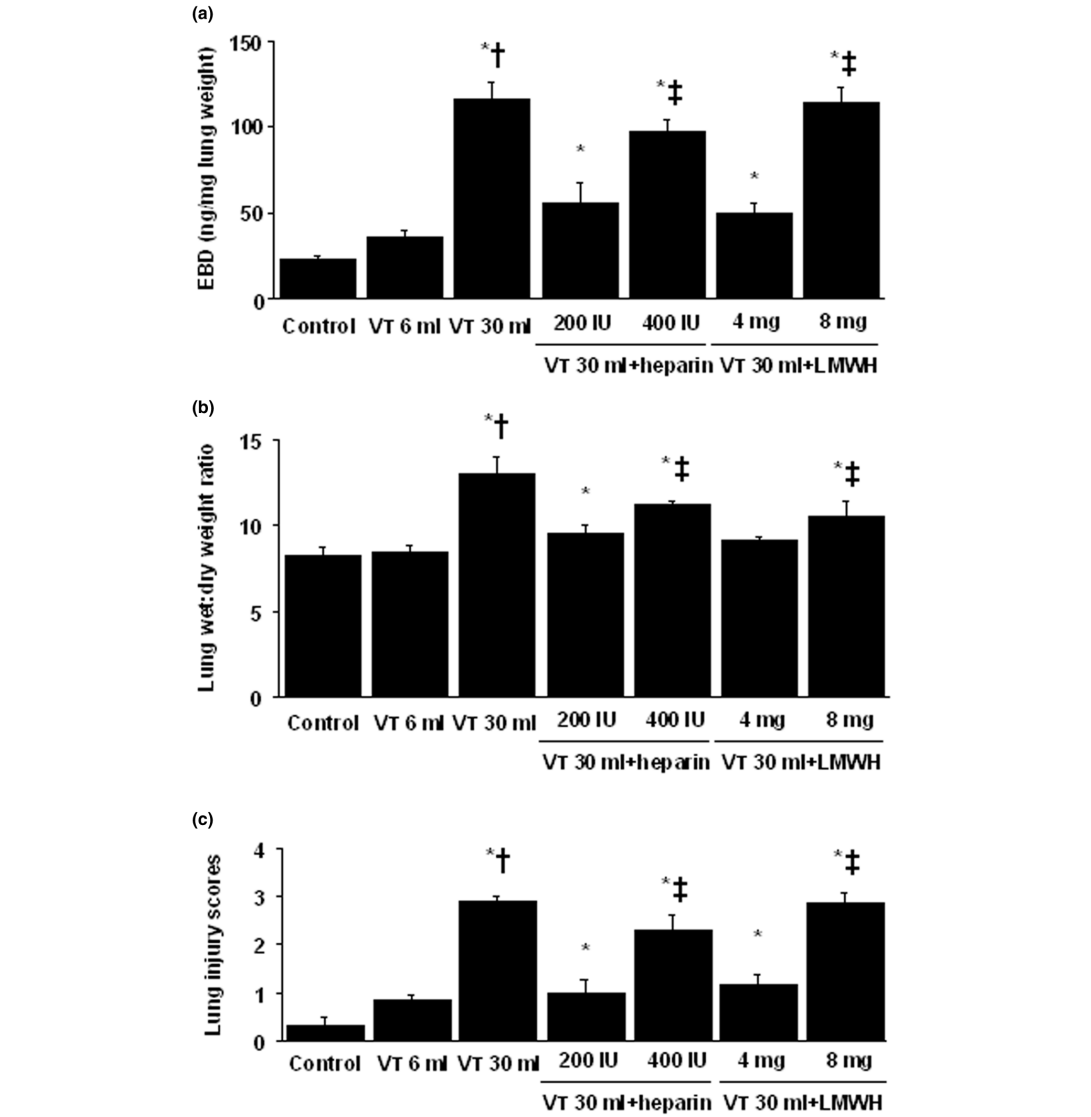
Unfractionated heparin and enoxaparin reduced lung stretch-induced microvascular leak, lung edema, and total lung injury. The mice were ventilated at a tidal volume (V_T_) of 6 ml/kg (V_T _6 ml) or 30 ml/kg (V_T _30 ml) for 5 hours with room air. Unfractionated heparin, 200 IU/kg or 400 IU/kg, and enoxaparin, 4 mg/kg or 8 mg/kg, was given subcutaneously 30 minutes before ventilation. **(a) **Evans blue dye (EBD) analysis of lung tissue (n = 6 per group). **(b) **Lung water measured by lung wet- to dry-weight ratio (n = 6 per group). **(c) **Total lung-injury scores of hematoxylin and eosin (H&E)-stained paraffin sections (n = 6 per group). * *P *< 0.05 versus control, nonventilated mice; † *P *< 0.05 versus all other groups; ‡ *P *< 0.05 versus 200-IU/kg or 4-mg/kg groups. H = unfractionated heparin; LMWH = enoxaparin.

To determine the effects of mechanical ventilation on changes of microvascular permeability and lung water in VILI, we measured lung EBD and the wet- to dry-weight ratio (Figure 1). The levels of lung EBD and wet- to dry-weight ratio significantly increased in mice receiving V_T _30 ml/kg mechanical ventilation compared with those of either V_T _6 ml/kg or control, nonventilated mice. No significant elevation was found in mice ventilated with V_T _6 ml/kg compared with control, nonventilated mice. The increases of microvascular leak and lung edema with V_T _30 ml/kg mechanical ventilation were significantly reduced by pharmacologic inhibition with unfractionated heparin and enoxaparin. Low-dose unfractionated heparin and enoxaparin were found to have better effects compared with those of high-dose unfractionated heparin and enoxaparin. Further to evaluate total lung injury, we measured alveolar congestion, hemorrhage, infiltration of neutrophils into the airspace or the vessel wall, and thickness of the alveolar wall. Increases of lung injury with V_T _30 ml/kg mechanical ventilation were significantly reduced by pharmacologic inhibition with unfractionated heparin and enoxaparin compared with those of control, nonventilated mice and mice ventilated at V_T _6 ml/kg.

### Inhibition of high-tidal-volume–induced neutrophil sequestration with unfractionated heparin and enoxaparin

**Figure 2:**
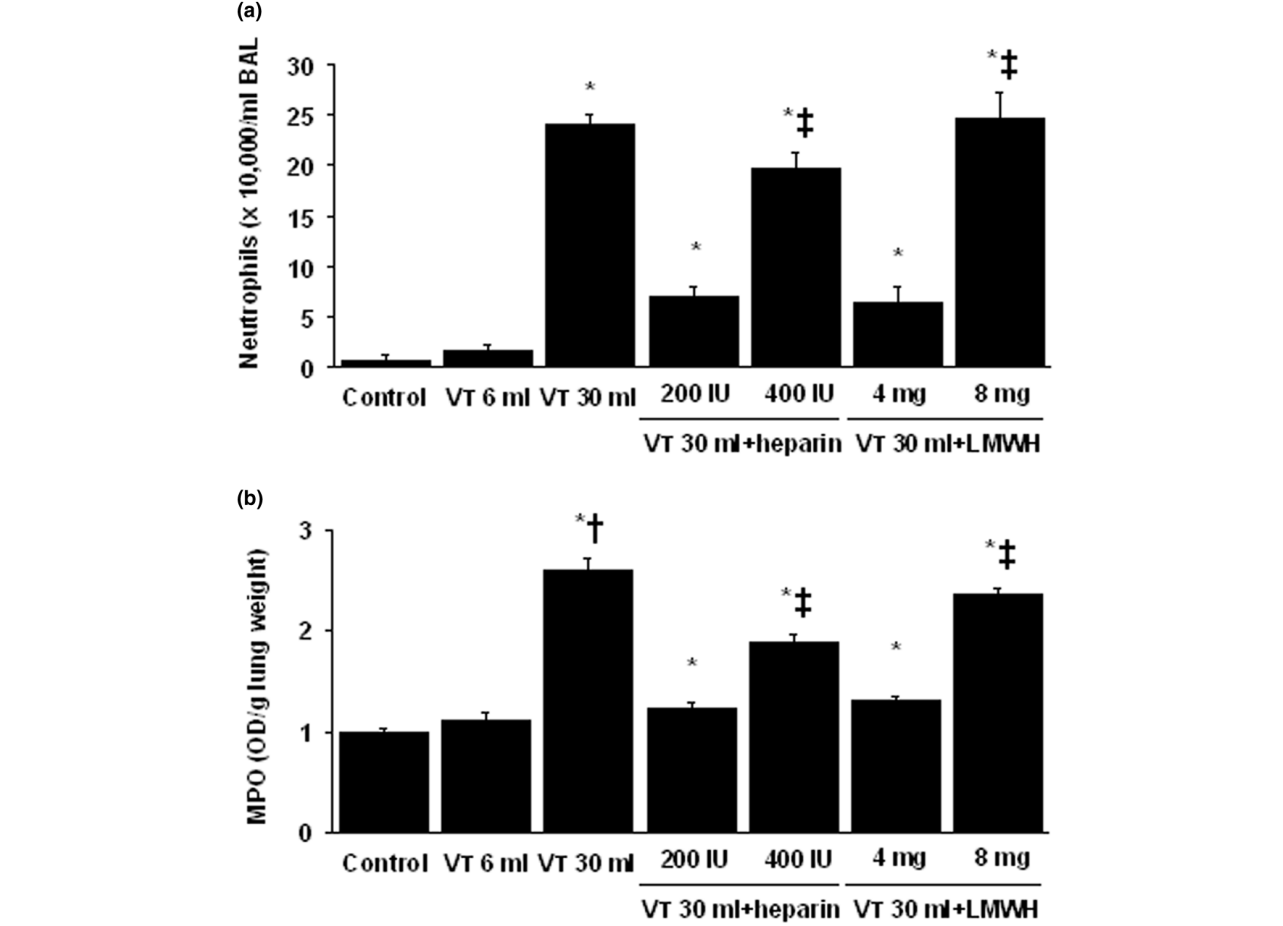
Unfractionated heparin and enoxaparin reduced lung stretch – induced neutrophil sequestration. The mice were ventilated at V_T _6 ml or V_T _30 ml for 5 hours with room air. Unfractionated heparin, 200 IU/kg or 400 IU/kg, and enoxaparin, 4 mg/kg or 8 mg/kg, was given subcutaneously 30 minutes before ventilation. **(a) **Neutrophils in bronchoalveolar lavage (BAL) fluid of lung tissue (n = 6 per group). **(b) **Myeloperoxidase (MPO) assay of lung tissue (n = 6 per group). *P < 0.05 versus control, nonventilated mice; † *P *< 0.05 versus all other groups; ‡ *P *< 0.05 versus 200-IU/kg or 4-mg/kg groups. H = unfractionated heparin; LMWH = enoxaparin.

Neutrophil counts were used to measure migration of neutrophils into the alveoli (Figure 2a). MPO assay was used to quantitate total lung neutrophils (*i.e.*, neutrophils marginated in the vasculature, located in the parenchyma and in the alveoli (Figure 2b). The neutrophil migration into lung lavage fluid and MPO levels were significantly elevated in mice after mechanical ventilation with V_T _30 ml/kg for 5 hours compared with mice ventilated with V_T _6 ml/kg and control, nonventilated mice. No significant elevation was found in mice ventilated with V_T _6 ml/kg compared with control, nonventilated mice. The increases in lung inflammation, as measured by neutrophils in the bronchoalveolar lavage fluid and lung MPO activity, with V_T _30 ml/kg mechanical ventilation were significantly reduced after pharmacologic inhibition with unfractionated heparin and enoxaparin. Low-dose unfractionated heparin and enoxaparin were found to have better effects compared with those of high-dose unfractionated heparin and enoxaparin.

### Inhibition of high-tidal-volume-induced PAI-1 mRNA expression and PAI-1 production with unfractionated heparin and enoxaparin

**Figure 3:**
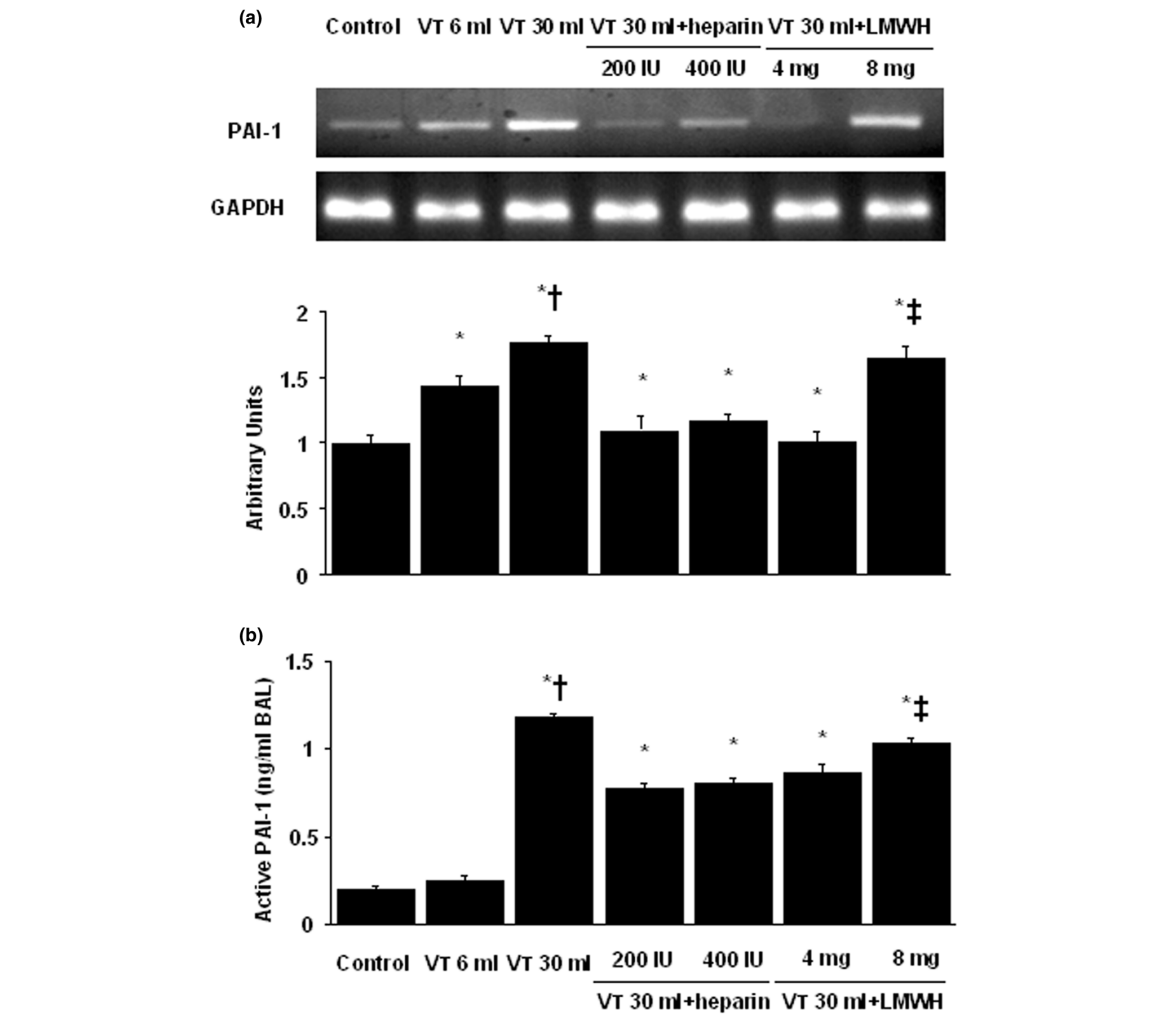
Unfractionated heparin and enoxaparin reduced stretch-induced plasminogen activator inhibitor-1 (PAI-1) mRNA expression and protein production. The mice were ventilated at V_T _6 ml or V_T _30 ml for 1 hour with room air. Unfractionated heparin, 200 IU/kg or 400 IU/kg, and enoxaparin, 4 mg/kg or 8 mg/kg, was given subcutaneously 30 minutes before ventilation. **(a) **Reverse transcription-polymerase chain reaction (RT-PCR) assay was performed for PAI-1 mRNA (a, top panel), glyceraldehydes-phosphate dehydrogenase (GAPDH) mRNA (a, middle panel), and arbitrary units (a, bottom panel) (n = 6 per group). Arbitrary units were expressed as the ratio of PAI-1 mRNA to GAPDH. **(b) **Active PAI-1 production in BAL fluid. *P < 0.05 versus control, nonventilated mice; † *P *< 0.05 versus all other groups; ‡ *P *< 0.05 versus 200-IU/kg or 4-mg/kg groups. H = unfractionated heparin; LMWH = enoxaparin.

To determine whether the increased neutrophil influx in mice receiving V_T _30 ml/kg mechanical ventilation was associated with upregulation of chemotactic factors for neutrophils, we measured PAI-1 mRNA expression and active PAI-1 protein production for 1 and 5 hours of mechanical ventilation, respectively (Figure 3). We found significantly increased ventilator-induced PAI-1 mRNA expression and PAI-1 protein production in the V_T _30 ml/kg mice compared with those of V_T _6 ml/kg or control, nonventilated mice. No significant increase in PAI-1 mRNA expression and PAI-1 protein production was observed in the V_T _6 ml/kg mice compared with control, nonventilated mice. This suggested that the increased expression of PAI-1 protein production might have been responsible for increased neutrophil infiltration. The increases in PAI-1 mRNA expression and PAI-1 protein production with V_T _30 ml/kg mechanical ventilation were significantly reduced after pharmacologic inhibition with unfractionated heparin and enoxaparin. Low-dose enoxaparin was found to have better effects compared with those of high-dose unfractionated heparin and enoxaparin.

### PAI-1-deficient mice reduced lung stretch-induced lung injury, neutrophil sequestration, mRNA expression, and PAI-1 production

**Figure 4:**
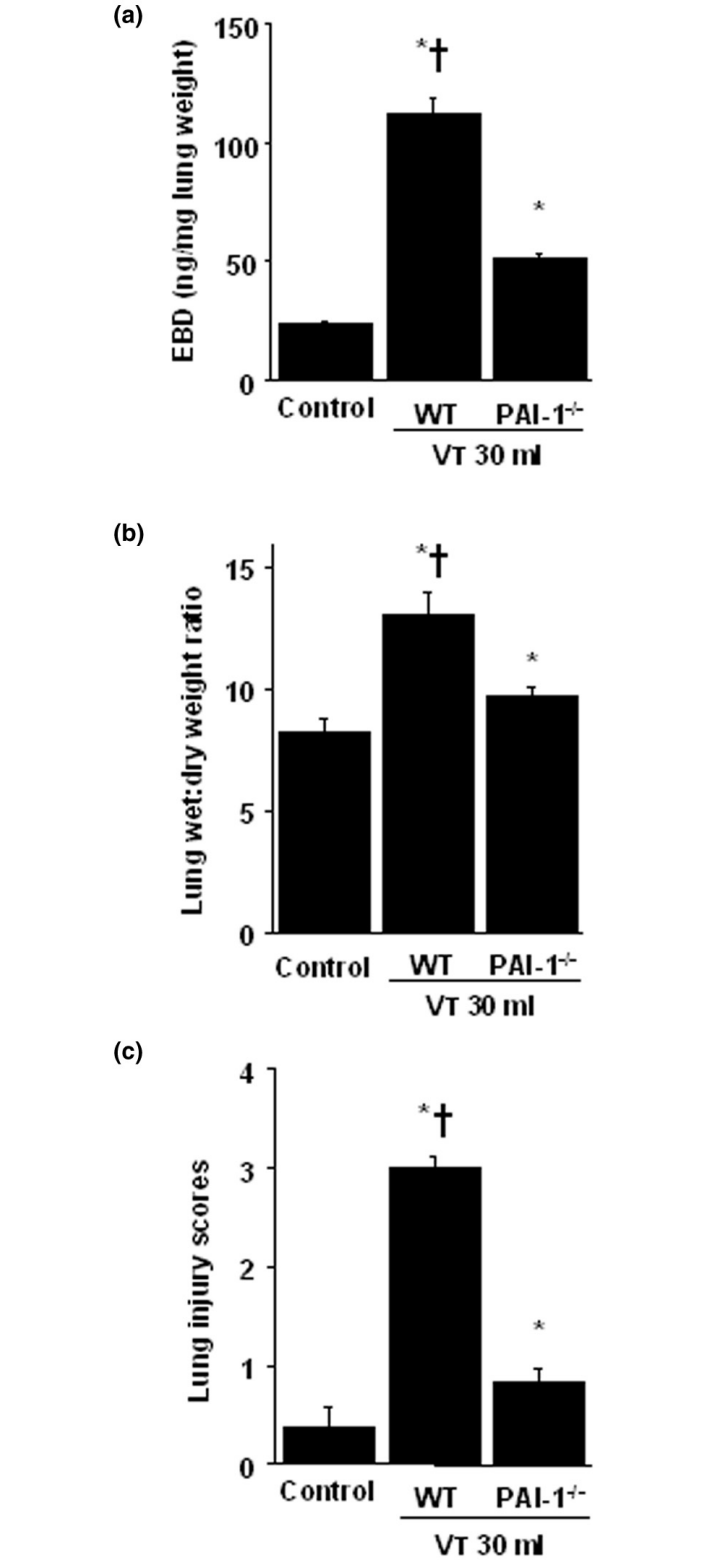
PAI-1–deficient mice reduced lung stretch–induced microvascular leak, lung edema, and total lung injury. Wild-type or PAI-1^-/- ^mice were ventilated at V_T _30 ml for 5 hours with room air. **(a) **EBD analysis of lung tissue (n = 6 per group). **(b) **Lung water measured by lung wet- to dry-weight ratio (n = 6 per group). **(c) **Total lung-injury scores of H&E-stained paraffin sections (n = 6 per group). * *P *< 0.05 versus control, nonventilated mice; † *P *< 0.05 versus PAI-1^-/- ^mice. EBD = Evans blue dye; PAI-1 = plasminogen-activator inhibitor-1.

**Figure 5:**
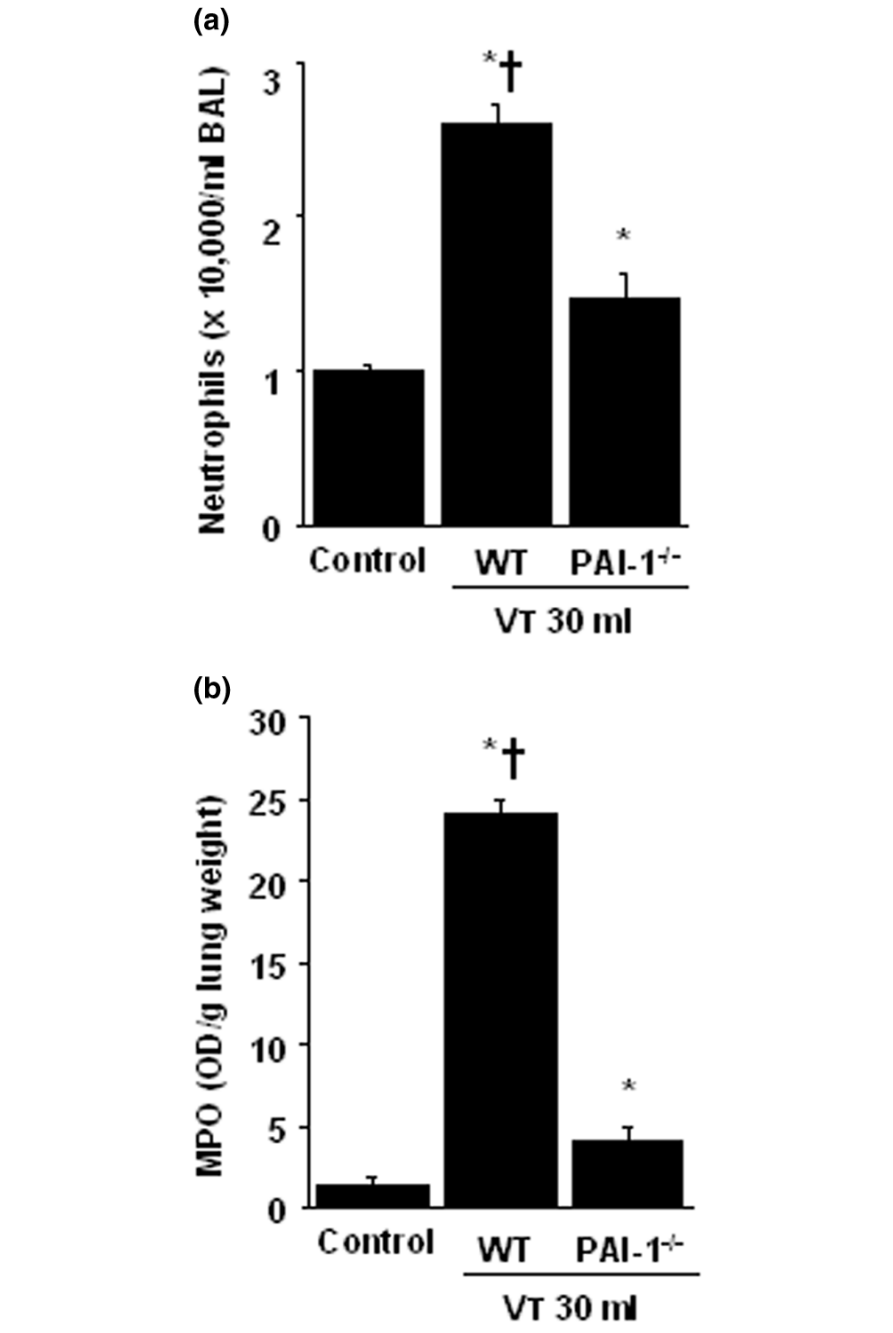
Plasminogen-activator inhibitor-1 (PAI-1)-deficient mice reduced lung stretch–induced neutrophil sequestration. Wild-type or PAI-1^-/- ^mice were ventilated at V_T _30 ml for 5 hours with room air. **(a) **Neutrophils in bronchoalveolar lavage fluid of lung tissue (n = 6 per group). **(b) **MPO assay of lung tissue (n = 6 per group). *P < 0.05 versus control, nonventilated mice; † *P *< 0.05 versus PAI-1^-/- ^mice.

**Figure 6:**
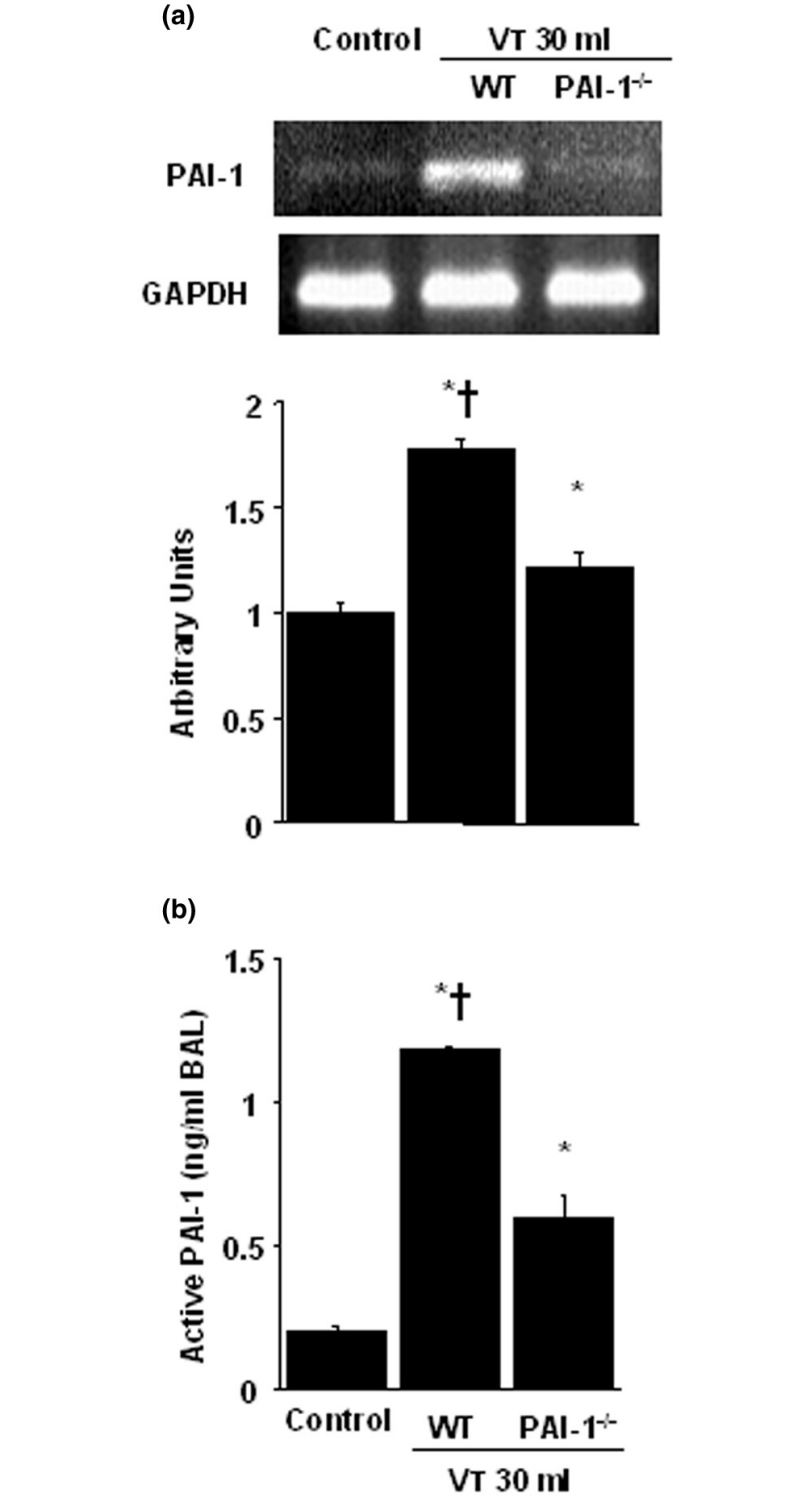
Plasminogen-activator inhibitor-1 (PAI-1)-deficient mice reduced lung stretch–induced mRNA expression and PAI-1 production. Wild-type or PAI-1^-/- ^mice were ventilated at V_T _30 ml for 1 hour with room air. **(a) **RT-PCR assay was performed for PAI-1 mRNA (a, top panel), glyceraldehydes-phosphate dehydrogenase (GAPDH) mRNA (a, middle panel), and arbitrary units (a, bottom panel) (n = 6 per group). Arbitrary units are expressed as the ratio of PAI-1 mRNA to GAPDH. **(b) **Active PAI-1 production in BAL fluid (n = 6 per group). *P < 0.05 versus control, nonventilated mice; † *P *< 0.05 versus PAI-1^-/- ^mice.

**Figure 7:**
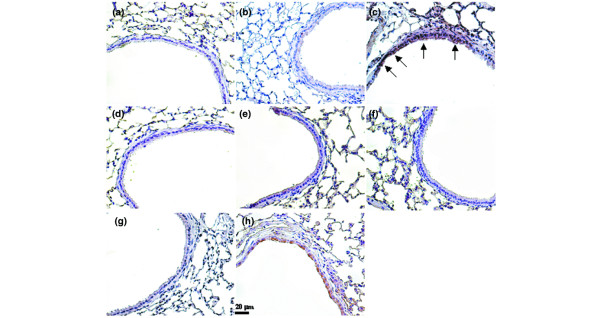
Unfractionated heparin, enoxaparin, and PAI-1-deficient mice reduced stretch-induced PAI-1 activation. Representative photomicrographs (×400) with PAI-1 staining of paraffin lung sections with immunohistochemistry were from mice ventilated at V_T _6 ml or V_T _30 ml for 5 hours with room air. Unfractionated heparin, 200 IU/kg or 400 IU/kg, and enoxaparin, 4 mg/kg or 8 mg/kg, was given subcutaneously 30 minutes before ventilation (n = 6 per group). **(a) **Control wild-type mice; **(b) **V_T _6 ml/kg wild-type mice; **(c) **V_T _30 ml/kg wild-type mice; **(d) **V_T _30 ml/kg wild-type mice pretreated with 200 IU unfractionated heparin; **(e) **V_T _30 ml/kg wild-type mice pretreated with 400 IU unfractionated heparin; **(f) **V_T_30 ml/kg PAI-1^-/- ^mice; **(g) **V_T _30 ml/kg wild-type mice pretreated with 4 mg/kg enoxaparin; and **(h) **V_T _30 ml/kg wild-type mice pretreated with 8 mg/kg enoxaparin. A dark brown diaminobenzidine (DAB) signal identified by arrows indicates positive staining for PAI-1 in the lung epithelium or interstitial, whereas shades of bluish tan signify nonreactive cells.

To determine the roles of PAI-1 activation in stretch-induced lung injury, we used PAI-1-deficient mice (Figures 4, 5, 6 and 7). The microvascular permeability, lung edema, neutrophil infiltration, PAI-1 mRNA expression, and PAI-1 protein production in mice ventilated at V_T _30 ml/kg for 5 hours were significantly reduced with PAI-1-deficient mice. This suggested that the reduction of high-tidal-volume-induced neutrophil influx and lung injury by unfractionated heparin and enoxaparin was dependent, in part, on the PAI-1 pathway. With immunohistochemistry, we further confirmed the effectiveness of unfractionated heparin and enoxaparin in the inhibition of PAI-1 production and the roles of PAI-1 involved in V_T _30 ml/kg ventilator-induced lung injury in bronchial epithelial cells (Figure 7).

## Discussion

High tidal volumes in normal animals have been used to mimic the overdistention of the less-injured and thus more-compliant areas of lung found in ARDS patients. Dreyfuss and co-workers [1] showed that hyperexpansion of the lung is the mechanism of pulmonary edema in VILI. Fibrinolytic activity is depressed in bronchoalveolar lavage fluids of patients with acute lung injury (ALI)/ARDS. Our previous animal model of lung fibrosis showed that simply overdistending lung tissue, in the absence of any other stimuli, causes dysregulation of fibrin turnover, but the mechanisms have been unclear [20]. As the most common cause of death in ALI/ARDS is multiple-organ-system failure, the systemic abnormalities of coagulation and fibrinolysis may be an important therapeutic target over the local pulmonary abnormalities. In a previous human study, ALI/ARDS was characterized by decreased plasma levels of protein C and increased plasma levels of PAI-1 that are independent risk factors for mortality and adverse clinical outcomes [21]. Heparin has been found to exert similar beneficial antiinflammatory activities as activated protein C [22]. Because LMWHs have a longer half-life, high bioavailability, predictable anticoagulant response, decreased risk of heparin-induced thrombocytopenia, and decreased risk of osteoporosis, we decided in this study to explore the role of LMWHs in the inhibition of pulmonary inflammation compared with unfractionated heparin [9]. In this mouse model of ALI, we found that high-tidal-volume ventilation in mice led to increased microvascular permeability, neutrophil influx, PAI-1 mRNA expression, production of PAI-1, and positive staining of PAI-1 in epithelium. Unfractionated heparin and enoxaparin regulated the decrease of fibrinolytic activity.

Alveolar PAI-1 levels have been found to be associated with a higher mortality rate in patients with ALI/ARDS [21]. Disturbances in coagulation and fibrinolysis, related to high pulmonary concentrations of PAI-1, have been demonstrated in patients with ALI/ARDS [23]. Others found that high-tidal-volume ventilation attenuated the fibrinolytic activity in rats pretreated with lipopolysaccharide (LPS), which was caused by increased production or release or both of PAI-1 [24]. In a previous study of injurious mechanical ventilation in rats, alveolar fibrinolytic capacity was suppressed because of local production of PAI-1 in lung epithelial cells triggered by TGF-β1 [6]. In our study, we found that high tidal volume increased lung inflammation demonstrated by changes of microvascular permeability, lung edema, neutrophil infiltration, and production of PAI-1. To define the role of PAI-1 production in stretch-induced neutrophil migration into the alveoli, PAI-1–deficient mice were used. In the setting of PAI-1 deficiency, neutrophils may remain inappropriately adherent to the extracellular matrix and unable to migrate into the alveolar compartment [25]. Shetty and co-workers [26] showed that regulation of uPA, uPA receptors, and PAI-1 occurred at the posttranscriptional level of mRNA stability regulated by p53 in lung epithelial cells. Similar to the results of others, we found transcriptional regulation of high-tidal-volume ventilation–induced PAI-1 mRNA expression, which suggested that other mechanisms may be involved in the regulation of PAI-1 production [11,27]. In addition to their anticoagulant activities, heparin and LMWH have been found to modulate the production and release of inflammatory cytokines [10,22,28]. In several studies, heparin and LMWH were shown to reduce lung edema and the chemotaxis of neutrophils related to the inhibition of C5a [5,22]. In an *in vivo *acute lung injury model in newborn piglets, heparin was shown to improve gas exchange and severity of hyaline membrane formation [28]. Heparin was shown to modulate migration of neutrophils, and the antiinflammatory effects of heparin depended on the degree of sulfation of the heparin molecule. Heparin also was found to be beneficial in the inhibition of PAI-1 production [5]. In a previous porcine acute lung injury model, LMWH was shown to attenuate neutrophil adhesion and TNF-α activity [10]. In our study, we found that both unfractionated heparin and enoxaparin reduced the high-tidal-volume-induced lung injury, neutrophil sequestration, and production of PAI-1. Previous study showed that low-dose heparin might have favorable effects on survival [22]. In our study, we found that low-dose unfractionated heparin and enoxaparin had better effects in reducing lung injury associated with an increase of neutrophil infiltration, but no significant differences in PAI-1 production were found between different heparin treatments. This inconsistency may be due to induction of PAI-1 by many proinflammatory factors and different cells, including platelets, fibroblasts, resident monocytes/macrophages, and endothelial and airway epithelial cells [4]. Higher doses of heparin/LMWH caused more bleeding complications (scoring of alveolar hemorrhage, as described in Methods: V_T _30 ml/kg with low-dose unfractionated heparin = 1.4 ± 0.4 versus V_T _30 ml/kg with high-dose unfractionated heparin = 2.5 ± 0.5; V_T _30 ml/kg with low-dose enoxaparin = 1.3 ± 0.3 versus V_T _30 ml/kg with high-dose enoxaparin = 3.1 ± 0.6) and induced proinflammatory effects because of the decrease of endothelial nitric oxide production and promotion of cell adhesion and platelet aggregation [22]. In a previous study of acute lung injury in human, PAI-1 antigen levels in both plasma and edema fluid were higher in patients with ALI, and differences in PAI-1 activity were concordant with levels of PAI-1 antigen [21]. Others showed that PAI-1 levels in plasma and pulmonary edema samples from patients with ALI/ARDS were associated with higher mortality rates [21]. In our study, we found that high-tidal-volume ventilation induced more increased PAI-1 activity in BAL fluid compared with those in plasma (fivefold versus twofold as compared with control, nonventilated mice). Unfractionated heparin and enoxaparin reduced high-tidal-volume-induced PAI-1 production in BAL fluid but not in plasma. This result and the studies of others suggested that PAI-1 production after injurious mechanical ventilation was in large part due to local activation of the fibrinolytic system [6,21,23]. Activation of coagulation and inhibition of fibrinolysis have been shown to be a local process because no systemic inhibition of fibrinolysis was found, and levels of coagulation and fibrinolysis markers were much higher in the pulmonary compartment than were the systemic levels [29].

Findings in other studies supported our results that neutrophil infiltration and the development of acute lung injury involved the PAI-1 pathway in an isolated mouse model of endotoxemia [25,30]. Previous *in vivo *study of LPS-pretreated mice showed that PAI-1 regulated neutrophil recruitment to inflammatory foci *via *mitogen-activated protein kinase pathways [25]. In an *in vitro *study of bovine aortic endothelium, others showed that Akt negatively regulated PAI-1 expression through the downregulation of P38/extracellular signal-regulated kinase1/2 pathways [31]. Experimental acute lung injury model of rat showed that local lung angiotensin II was involved in the pathogenesis of disordered coagulation of PAI-1 production in VILI [11]. In the study of patients with ARDS, others showed that alveolar epithelial cells can activate protein C, and the ability to activate protein C was diminished in response to exposure to proinflammatory cytokines, including PAI-1 [32]. Previous study also showed that markers of dysregulated coagulation and fibrinolysis, such as IL-8, intercellular adhesion molecule 1, and protein C, are predictive of clinical outcomes in patients with ALI/ARDS [30]. Heparin may inhibit pulmonary coagulation by affecting other cell types in the lung, including endothelial cells and fibroblasts. Both heparin and LMWH have been shown to inhibit the activation of nuclear factor-κB and attenuated endothelial dysfunction by enhancing nitric oxide [22]. Heparin and LMWH can also inhibit neutrophil adhesion to P-selectin *in vitro *and modulate the hemodynamic effects of platelet-activating factor (PAF) and thromboxane B_2 _biosynthesis *in vivo*, suggesting the mechanism of altering endothelial P-selectin/PAF [10]. With immunohistochemistry, we confirmed the effects of unfractionated heparin and enoxaparin on the increase of PAI-1 staining in mice ventilated at V_T _30 ml/kg in the bronchial epithelia but not in endothelial cells. The discrepancies of cell types involved may be due to the different physical forces of mechanical strain and immunohistochemistry method limitations. Activated protein C associated with high mortality in patients with ARDS was not measured in this study because of unavailability of a commercial immunoassay kit for mice.

## Conclusions

Although the ARDS network trial demonstrated that low- is safer than high-tidal-volume ventilation, these findings have been questioned. In the combined rat model of VILI and acid aspiration, V_T _3 ml/kg was more protective than 6 ml/kg, so even very low V_T _of 6 ml/kg can cause lung injury. The National Heart, Lung and Blood Institute working group on acute lung injury identified examination of the biology of stress-induced injury to the lung in health and disease as a fertile area of future research, because ventilation-induced release of cytokines may lead to systemic translocation and multisystem organ failure [33,34]. With an *in vivo *mouse model of acute lung injury, we demonstrated that high-tidal-volume mechanical ventilation increased microvascular permeability, neutrophil influx, lung PAI-1 mRNA expression, production of active PAI-1, and the deleterious effects were attenuated by low-dose unfractionated heparin or enoxaparin treatment. These results imply that dysregulated coagulation and fibrinolysis are involved in the pathogenesis of VILI and that the protective mechanism of heparin/LMWH attenuation of VILI may be related to a reduction in PAI-1. In addition to the lung-protective strategy used in the management of patients with ALI, our data added to the understanding of the effects of mechanical forces in the lung and may permit possible strategies directed at preventing VILI to be instituted early in the course of the disease process.

## Key messages

• High-tidal-volume ventilation increased plasminogen activator inhibitor-1 in acute lung injury.

• Inhibition of plasminogen activator inhibitor-1 by unfractionated heparin and enoxaparin may offer new treatment options for patients with severe ARDS.

## Abbreviations

ALI: acute lung injury; ARDS: acute respiratory distress syndrome; BAL: bronchoalveolar fluid; DAB: diaminobenzidine; EBD: Evans blue dye; ES: embryonic stem cells; GAPDH: glyceraldehyde-phosphate dehydrogenase; H&E: hematoxylin and eosin; IHC: immunohistochemistry; LPS: lipopolysaccharide; LMWH: low-molecular-weight heparin; MIP-2: macrophage inflammatory protein-2; MPO: myeloperoxidase; PaCO_2_: arterial carbon dioxide pressure; PaO_2_: arterial oxygen pressure; PAF: platelet-activating factor; PAI-1: plasminogen activator inhibitor-1; RT-PCR: reverse transcription-polymerase chain reaction; TGF-β1: transforming growth factor-β1; TMB: 3,3', 5,5'-tetramethylbenzidine; TNF-α: tumor necrosis factor-alpha; tPA: tissue-type plasminogen activator; uPA: urokinase-type plasminogen activator; VILI: ventilator-induced lung injury; V_T_: tidal volume.

## Competing interests

The authors declare that they have no competing interests.

## Authors' contributions

L-LF and L-SK collected and analyzed the data. Q-DA, L-HC, H-CC, and T-YH, reviewed and coordinated the study.
